# From the Lab to the Farm: An Industrial Perspective of Plant Beneficial Microorganisms

**DOI:** 10.3389/fpls.2016.01110

**Published:** 2016-08-04

**Authors:** J. Jacob Parnell, Randy Berka, Hugh A. Young, Joseph M. Sturino, Yaowei Kang, D. M. Barnhart, Matthew V. DiLeo

**Affiliations:** BioAg, Novozymes, DurhamNC, USA

**Keywords:** biofertility, biocontrol, commercialization, agricultural products, food security

## Abstract

Any successful strategy aimed at enhancing crop productivity with microbial products ultimately relies on the ability to scale at regional to global levels. Microorganisms that show promise in the lab may lack key characteristics for widespread adoption in sustainable and productive agricultural systems. This paper provides an overview of critical considerations involved with taking a strain from discovery to the farmer’s field. In addition, we review some of the most effective microbial products on the market today, explore the reasons for their success and outline some of the major challenges involved in industrial production and commercialization of beneficial strains for widespread agricultural application. General processes associated with commercializing viable microbial products are discussed in two broad categories, biofertility inoculants and biocontrol products. Specifically, we address what farmers desire in potential microbial products, how mode of action informs decisions on product applications, the influence of variation in laboratory and field study data, challenges with scaling for mass production, and the importance of consistent efficacy, product stability and quality. In order to make a significant impact on global sustainable agriculture, the implementation of plant beneficial microorganisms will require a more seamless transition between laboratory and farm application. Early attention to the challenges presented here will improve the likelihood of developing effective microbial products to improve crop yields, decrease disease severity, and help to feed an increasingly hungry planet.

## Introduction

The alarm cry of impending global food shortages is not new. Over the centuries figures such as Tertullian, Townsend, Malthus, and Ehrlich (Hardin, 1998; Alexandratos and Bruinsma, 2012) have warned of dire consequences of the inability of Earth’s capacity to sustain its growing population (Ehrlich and Ehrlich, 1990). Each time, crisis has been averted due to technological advances in plant breeding, fertilization, crop protection and agronomic management. For example, over the past 50 years the human population of our planet has doubled, and the need for increased food production was met by the application of new technologies, such as the discovery of the Haber-Bosch process (Erisman et al., 2008), and agronomic management strategies. Although they contributed to staving widespread famine and saving billions of lives, novel and complementary solutions are needed to continue to improve crop yield. As we face our next challenge, it is critical that we continue to discover new sustainable cropping system solutions to produce more with fewer resources.

By the year 2050, the global population is expected to reach 9.6 billion which has been estimated as our planet’s maximum capacity (Wilson, 2003). This increase in population will require at least double our current agricultural production, despite the challenges with current resource requirements and a decline in arable land (Bruinsma, 2009). Similar to the green revolution, in order to ensure global food security for a growing population we need to devise enhanced cropping systems that maximize productivity while minimizing the resources required. In most agricultural lands, maximizing yield requires additional inputs to maintain productivity and crop yields. These additions include both phosphorus and nitrogen as fertilizer, as well as pesticides that help control invasive weeds, pathogens and insects. Farmers could benefit from new sustainable products to boost or maintain yields, often under increasing environmental stresses (Baulcombe et al., 2009). While chemistries and trait development remain critical in developing stress tolerance and pathogen resistance programs of agriculture, the application of microbial products is now considered a valuable addition to precision agriculture (Berg, 2009; Bhattacharyya and Jha, 2012).

Microbial products have been used commercially in global agriculture for over 120 years (Nobbe and Hiltner, 1896; Deaker et al., 2004), but have recently received increased attention. There are currently over 149 registered microbial strains for agricultural products (Copping, 2009). A recent special publication by the American Society for Microbiology suggested that microbes may be, at least in part, a sustainable solution to increasing agricultural production and outlined current shortcomings of microbes in helping to feed the world (Reid and Greene, 2013). The market for commercial biofertility inoculant and biocontrol products in 2012 was valued at over $1 billion US dollars (USD) and is expected to exceed $7 billion USD by 2019, increasing at a double digit compound annual growth rate (CAGR) between 2013 and 2019 (Transparency Market Research, 2014). Major growth drivers include growing consumer interest in organic crops, reducing synthetic products, and the economic potential in emerging markets such as China (Transparency Market Research, 2014). Despite the benefits and potential of agricultural microbial products, a recent spotlight on plant yield promoting bacteria pointed out that “The scientific literature abounds with many potentially highly useful strains that did not appear on the commercial market” (Bashan et al., 2014). In a 30 year span ending in 2002, an estimated 72% of biocontrol business ventures failed (Glare et al., 2012). Most often, failures result from underestimating costs associated with developing and marketing microbial products (CPL, 2006, Biopesticides). The incongruence between effective microbial strains and successful agricultural products suggests a need to address obstacles that may not be anticipated.

Microbes will certainly play a role in revolutionizing agriculture over the next several decades to help meet the demands of a growing population. Promising agricultural products include organisms that increase crop yield through enhanced nutrient update by plants (inoculants), and organisms that reduce crop loss due to pests (biocontrol). While timely and extremely valuable, the American Society for Microbiology report (Reid and Greene, 2013) focuses primarily on what Bashan et al. (2014) call the ‘research facility’ side of product development and omits important characteristics of the ‘industry’ role. This review provides an industrial perspective on the current state of these types of microbial products. Also, in an effort to help maximize the number of strains that make a practical impact on agriculture, some of the challenges involved with taking a successful laboratory strain and making a viable commercial product are discussed.

## Biofertility Inoculants

Deployment of microbes to enhance crop productivity by boosting the availability of key nutrients is a concept widely referred to as biofertility. Biofertility inoculants as defined above is not a new concept, and the commercial application of inoculants dates from the launch of a bacterial product for legumes called “Nitrogin” by Nobbe and Hiltner (1896) and Sahoo et al. (2013). In the late 1940s, Timonin (1948) disclosed bacterial products termed “Alnit” to augment the productivity of non-legume crops. The market for commercial biofertility inoculants in 2012 was valued at $440 million USD and is expected to exceed $1 billion USD by 2019, growing at a CAGR of 13% between 2013 and 2019 (Transparency Market Research, 2014).

The most limiting soil nutrients for plant growth are nitrogen and phosphorus (Schachtman et al., 1998). Although many soils contain ample quantities of these nutrients, most are not readily accessible for plant growth (Rai, 2006). Consequently, microbial products have been developed to increase the availability of nitrogen or phosphorus to crops (Vance, 2001), thereby maximizing the efficient, sustainable use of nutrients.

### Nitrogen-Fixing Microbes

Nitrogen is the most critical nutrient for plant growth, and perhaps the most recognizable example of biofertility inoculants are the rhizobia which fix atmospheric nitrogen in nodules of legume crops. This diverse group of bacteria comprises some of the most intensely investigated microbes owing to their value as inoculants. Despite their taxonomic diversity, all rhizobia establish symbiotic interactions with their host plant via highly conserved mechanisms which have been reviewed extensively (Alexander, 1984; Weidner et al., 2003; Zahran, 2009; Terpolilli et al., 2012). Legume crops are grown on an estimated 250 million hectares globally and fix roughly 90 million metric tons of atmospheric nitrogen annually (Zahran, 2009).

Effective rhizobial products exhibit high rates of nitrogen fixation and compete successfully with less efficient indigenous rhizobia populations to colonize and form nodules on target host plants. Successful commercial production of rhizobia required the ability to produce the organisms in large quantities and enable a long-term shelf life. Unfortunately, many microbial products fall short in the latter specification leading to overall poor performance in the field. In the 1980s and 1990s many rhizobial products showed poor efficacy (Catroux et al., 2001). However, over the past decade both quality standards and performance of these products have improved substantially, and several marketed products have been shown to affect consistent improvements in yields of legume crops. Nitrogen-fixing products sold today contain substantially higher numbers of viable organisms per gram than those from earlier decades. Additionally, improved product formulations have resulted in enhanced stability (Grooms, 2008). In tests with inoculated soybeans, Beuerlein (2008) reported yield improvements averaging approximately 120 kg per hectare.

**FIGURE 1:**
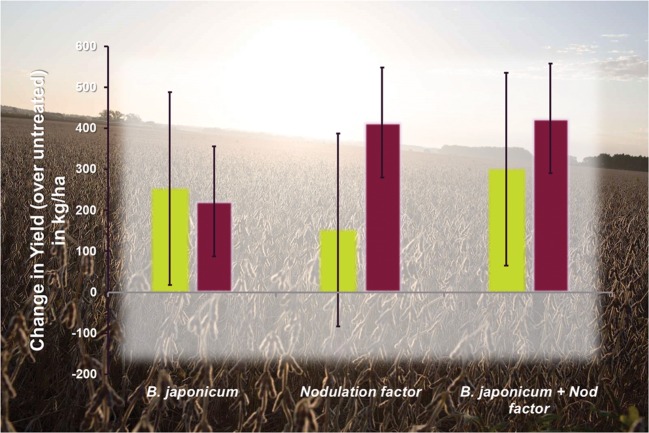
**Performance of *Bradyrhizobium japonicum*, a nodulating factor (LCO), and the combination of *B. japonicum* and nodulating factor (Optimize^®^) in field trials.** Field trials occurred in pristine soil (no previous soy; violet), and soils with previous soy crops (green). Error bars represent least significant difference at 95% (adapted from Smith et al., 2015).

Soybeans contain 37–45% protein by weight, and thus, a 3600 kg ha^-1^ crop requires 136 kg of nitrogen (Beuerlein, 2008). To illustrate the impact of rhizobial products on soybean yields, products sold by the Monsanto BioAg Alliance (Optimize^®^) (Monsanto BioAg Alliance, 2015e), BASF (Vault^®^), ABM (Excalibre^TM^), and MycoGold^TM^ are discussed. In addition to live *Bradyrhizobium* cells, Optimize^®^ for soybeans contains lipochitooligosaccharide, a molecule that enhances the soil microbial environment^1^ Seeds treated with Optimize^®^ consistently show an increase in yield over untreated controls (**Figure 1**). Similarly, Vault^®^ is a seed treatment consisting of *Bradyrhizobium* and a patented rhizobial enhancer (Basf-Corporation, 2015). ExcalibreSA^TM^ is a blend of Bradyrhizobia^2^, and MycoGold^TM^ blends *Bradyrhizobium* with biostimulants and other microbes^3^ The use of bioinoculants on soybean crops consistently provides a 4:1 return on investment.

In addition to the nodule-forming rhizobia which establish nitrogen-fixing symbioses in legumes, there are numerous species of non-legume nitrogen fixing bacteria that associate with agriculturally important crops (Rai, 2006). Among these, members of the genera *Azospirillum* MicroAZ-ST^TM^ (TerraMax^4^), and Mazospirflo-2 (Soilgro; Owen et al., 2015), *Azotobacter* Bio-N^TM^ (Agriculture Solutions^5^), and *Gluconacetobacter* have attracted interest, because they are root-colonizing and exhibit the potential to transfer fixed nitrogen to the plants with which they associate. Non-legume nitrogen fixing bacteria have been shown to increase yield of various crops including sunflower, carrot, oak, sugar beet, sugar cane, tomato, eggplant, pepper, cotton, wheat, and rice (Bashan et al., 1988; Bashan and Holguin, 1997). In a review summarizing 20 years of global field trials, Okon and Labandera-Gonzales (1994) reported that in 60–70% of the trials, inoculation with various *Azospirillum* strains increased crop yields by 5–30%. Another extensive, multi-year study conducted by Diaz-Zorita et al. (2012) showed that on-seed inoculation increased wheat and maize yields by 244 kg ha^-1^ (3.9 bu ac^-1^) and 514 kg ha^-1^ (8.2 bu ac^-1^), respectively. In addition to nitrogen fixation, some *Azospirillum* species are capable of producing plant growth-promoting compounds which may play a role in their mode of action (Okon et al., 2015). Non-leguminous nitrogen fixing bacteria also manifest other plant-beneficial traits such as remediation of soils polluted with heavy metals (Ullah et al., 2015) and confer enhanced tolerance in plants to abiotic stresses such as drought (Vargas et al., 2014).

### Phosphate Solubilizing Microbes

Compared with other soil nutrients, phosphorus is the least mobile and is usually in a relatively unavailable form for plant uptake. Next to nitrogen, this nutrient is the second most important nutrient in crop production and is traditionally applied in the form of chemical fertilizers or manure. The world’s supply of rock phosphate is expected to be largely depleted in the next few decades (Gilbert, 2009; Scholz et al., 2013). With China, India, and the US as the major users of rock phosphate and 70% of known deposits located in China, Russia, Morocco, and the US, the long term sustainability of current phosphate resources is debated. To ensure the most efficient use of limited supplies of rock phosphate fertilizer and circumvent future shortages, phosphorus-solubilizing microorganisms have been developed to enhance the nutrition of crops in a sustainable manner.

Ironically, the total amount of phosphorus in soils may be high, but it is usually present in forms that are unavailable for plant growth. These comprise both organic and inorganic pools, of which 20–80% can be found in organic forms that include phytic acid (inositol hexaphosphate) as a major component (Richardson, 1994). The largest fraction of inorganic phosphate in soil resides in complexes with metals (particularly Ca, Al, and Fe) (Richardson, 2001). Soil microbes that liberate phosphate from organic and inorganic pools have been promoted as products that effectively mobilize phosphate from poorly available sources in soil and reduce the application of rock phosphate fertilizer. Products such as these are expected to show rapid commercial growth over the next few years. While the genetic and biochemical components underlying the mechanisms of phosphate-liberation by these organisms have not been as extensively studied as nitrogen fixation, excretion of organic acids and synthesis of phosphate-scavenging enzymes such as phytases have been implicated in their modes of action (Richardson, 2001).

Pools of insoluble phosphate in metal complexes can be made available to plants through the action of phosphorus-solubilizing microorganisms. Improved crop yields resulting from the application of phosphorus-solubilizing organisms in the field have been reported (Pradhan and Sukla, 2005), notably *Bacillus* (Symbion-P^®^) and *Pseudomonas* among bacterial genera, and *Aspergillus* and *Penicillium* are among the most important fungal taxa. In comparing characteristics of phosphorus-solubilizing bacteria and fungi, it has been reported that fungi exhibit greater solubilizing activity than bacteria (Nahas, 1996). *Penicillium bilaiae* is a fungus present in the commercial product Jumpstart^®^ marketed by the Monsanto BioAg Alliance (2015a). The organism solubilizes soil phosphorus by a mechanism that involves secretion of citric and oxalic acids (Cunningham and Kuiack, 1992). A recent publication by Leggett et al. (2015) summarized the findings of a large multi-year field study to assess the yield responses of maize to inoculation with JumpStart^®^. Rigorous statistical analyses of both large and small test plots revealed significant yield increases in 66 of 92 (72%) small plots and 295 of 369 (80%) large plots (**Table 1**). These results strongly suggest a significant impact on maize yields as a result of the fungus *P. bilaiae*. The lack of successful commercial phosphate-solubilizing inoculants has been noted (Leggett et al., 2001) and attributed to plant or environmental incompatibility.

**Table 1:** Summary of small and large plot field trials to measure maize yield response to inoculation with the phosphorus-solubilizing fungus *Penicillium bilaiae* (adapted from Leggett et al., 2015).

Trials	Sample size, *n*	Yield increase (kg/ha ± SE)	Increase %
Small plot	92	169 ± 2.8	1.8
Large plot	92 369	326 ± 1.6	3.5


Another group of phosphate solubilizing microorganisms are arbuscular mycorrhizal fungi (AMF) that are able to form a network of hyphae that interact with the plant roots to improve nutrient transport and protect the plant against pathogens and some forms of abiotic stress (Porcel et al., 2012; Hodge and Storer, 2015). Most of the vascular plants on Earth form an association with AMF (Smith and Read, 2008); they are ubiquitous and ecologically important for soil health. Within the AMF, the most widely used products in agriculture usually belong to the phylum Glomeromycota (Owen et al., 2015) and have been shown to increase P uptake. Some of the examples of AMF products include Mycormax^®^ (JH Biotech^6^), BEI (BioOrganics^TM^^7^), BioGrow Endo (Mycorrhizal Applications^8^), and VAM (Microbesmart^9^).

### Products Containing Multiple Biofertility Microbes

**FIGURE 2:**
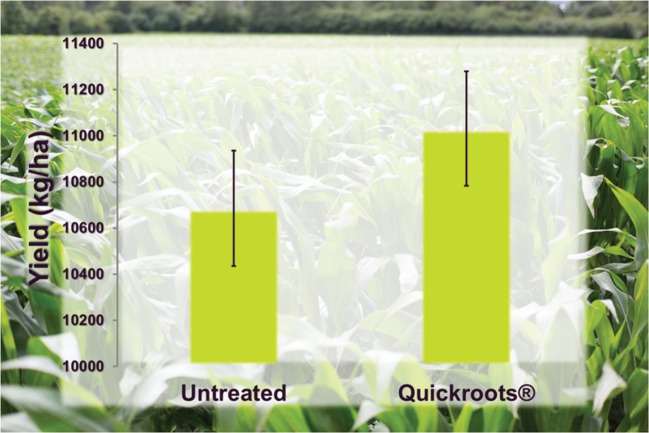
**Performance of QuickRoots^®^ (*Bacillus amyloliquefaciens* plus *Trichoderma virens*) product compared with untreated corn seeds in small plot trials (*N* = 104).** Error bars represent Standard Error. Yield values are significantly different (two-tailed *t*-test < 0.001).

Interestingly, a few commercial products have emerged that take advantage of combining different biofertility products. One such product, marketed under the trade name QuickRoots^®^, is sold by the Monsanto BioAg Alliance (2015b). This product contains a patented combination of the *Bacillus amyloliquefaciens* and the filamentous fungus *Trichoderma virens* (Monsanto BioAg Alliance, 2015c,d). Both of these organisms are known to liberate bound phosphate making this nutrient more available to plant roots (Fan et al., 2011; Akladious and Abbas, 2012; Molla et al., 2012; Lamdan et al., 2015), and the combination purportedly imparts increased availability of nitrogen, phosphorus and potassium in soil resulting in expanded root volume for enhanced yield potential^10^ Field trial data with QuickRoots^®^ applied to corn shows a positive yield ranging from 220 to 500 kg ha^-1^ increase representing a 2:1 to 5:1 return on investment (**Figure 2**)^numfont 10^. Lastly, the combination of these two organisms may also enhance favorable interactions of plant roots with mycorrhizal fungi in the soil (Johnson, 2013, 2015). Other examples of mixed products include Excalibre-SA (ABM), which combines *Trichoderma* with *Bradyrhizobium* for soy^11^, and BioGrow Endo (Mycorrhizal Applications) combines AMF and *Trichoderma*^12^

## Biocontrol Organisms

Plant diseases and pests are among the largest contributors to crop losses worldwide, with an estimated 27–42% in production systems and potential losses of 48–83% in the absence of crop protection (Oerke and Dehne, 2004). The use of biological organisms to control plant disease (biocontrol) could potentially augment the use of synthetic pesticides (e.g., residue and resistance management). Despite clear enthusiasm around the potential for biocontrol microbes, challenges still exist in efficacy, field performance, and cost. In this section, the role of biocontrol in plant pest management from an industry perspective is addressed. We focus on both the scientific and production strategies necessary to bring biocontrol products to market, and highlight a few examples of commercially available biocontrol strains.

Biocontrol research has received a lot of attention in recent years, and there are many well documented examples of biocontrol microbial activity in scientific literature (Glare et al., 2012; Junaid et al., 2013; Bardin et al., 2015; Pelizza et al., 2015), however, synthetic pesticides still dominate the commercial market (Elad, 2003). Only an estimated 3.5% of the global pesticide market is represented by biocontrol products (valued at 1.6 billion USD in 2009) (Lehr, 2010). In North America and Western Europe, biocontrol markets have been estimated to be $594.2 million in 2009 and $1.09 billion in 2015 (Frost and Sullivan, 2009). Although challenged by issues of performance and cost, it is clear that the biocontrol market is growing rapidly. Estimates have proposed a 15.6% CAGR, resulting in over 7% global market shares in 2014 (Lehr, 2010; Glare et al., 2012). Regardless of how size estimates are made, all indications point to continued growth for the biocontrol market, well beyond that predicted for the synthetic pesticide market (Glare et al., 2012).

Historically, early sales within the biocontrol market consisted predominately of a single product type containing *Bacillus thuringiensis* (Bt) targeted against lepidopterans (e.g., cabbage worms and gypsy moth). In 1990, over 90% of biocontrol sales corresponded to Bt-related products, with a total market of approximately $120 million USD (Rodgers, 1993), although other biocontrol products such as entomopathogenic nematodes have played a key role (Shapiro-Ilan and Gaugler, 2002). After 2 decades, the estimated total sales for microbial-based biocontrols was close to $400 million USD with just over 50% of sales corresponding to Bt-related products (Glare et al., 2012). The geographical distribution of biocontrol sales has changed dramatically over the last two decades to cover a broader global market and a greater number of agricultural crops (Rodgers, 1993; Glare et al., 2012). These trends suggest that the geography, market sectors, major arable crops, and diversity of microbial strains all continue to expand. Major drivers for growth in biocontrol use include growing consumer interest for products in emerging markets such as China and India.

Broad adoption of biocontrol products into mainstream agriculture requires advances in technology, increased understanding of the biology and ecology of active organisms, and cost effective, efficacious products. Industry concerns generally focus on production, formulation, and delivery when commercializing a biocontrol product (Fravel, 2005). In addition to these attributes, industry must consider aspects of product registration, intellectual property, and an understanding growers needs. Finally, aspects of efficacy, persistence, and mode of action (biology) must be considered when developing an effective biocontrol product.

### Biology of Biocontrol

Biocontrol agents are broadly classified as preparations either derived-from or containing living microorganisms that can prevent or suppress pests like pathogens, insects, and weeds. Biocontrol agents can include living microbes (bacteria, fungi, nematodes, viruses and protozoa), bioactive compounds such as secondary metabolites (e.g., spinosads and avermectins), or naturally derived material such as plant extracts (Kiewnick, 2007; van Lenteren, 2012). Pest damage prevention by biocontrol agents is based on several mechanisms that may involve antibiosis, competition for space and nutrients, mycoparasitism, enzymatic activity, and induced resistance (Lo, 1998). These modes of action are certainly not exclusive, and biocontrol agents likely enlist a combination of activities when counteracting disease.

As previously mentioned, industrial application of biocontrol microbes will require a deeper understanding of the biology of the microbe, the targeted pest or pathogen, and interactions with host plants, other microbes, and the environment. Drivers of microbe communities in the rhizosphere, for example, involve soil type and plant genotype (Berg and Smalla, 2009; de Bruijn, 2013), whereas the phyllosphere microbiome is influenced by plant genotype and environmental factors like humidity, ultraviolet light, and geographic location (Vorholt, 2012; Rastogi et al., 2013). Understanding these ecological differences is critical when making decisions about product development and commercial application. In an illustration of abiotic effects, biocontrol efficacy by nonpathogenic *Fusarium oxysporum* was significantly affected by both temperature and light (Larkin and Fravel, 2002). In another example of biological complexity, Erlacher et al. (2014) found that shifts in lettuce microbe communities caused by pathogen infection (*Rhizoctonia solani*) were offset by the biocontrol agent *Bacillus amyloliquefaciens* FZB42. Such selective compensation of pathogen impact by a biocontrol strain suggests a novel mode of action and highlights the complexity of biocontrol within a plant–microbe ecosystem. Understanding how biocontrol microorganisms interact with one another represents another biological challenge for product development. Co-inoculation of *Trichoderma viride* strain GB7 and *Serratia plymuthica* strain 3Re4-18 resulted in greater biocontrol efficacy against *R. solani* in lettuce, compared to application of single strains (Grosch et al., 2012). However, combined biocontrol application also had a more pronounced impact on the microbial community structure at large (Grosch et al., 2012). These studies highlight the complex and fluid interactions between plant, pathogen, biocontrol agent, microbe community, and the environment. To commercialize effective biocontrol microbes as products, industries will need to invest in fundamental and early development research surrounding these biological questions. This will require deeper partnerships within industry as well as greater communication with academic (public and private) and government research organizations.

### Screening for Biocontrol

Commercialization of a successful biocontrol product ultimately depends on the availability and isolation of candidate microbes. This screening process involves isolation from a particular environment and early trials to characterize a microbe’s biocontrol capability. While no single screening method is optimal for all biocontrol endeavors, a logical strategy should be followed based upon the pathosystem (plant-pathogen-environment) of interest (Fravel, 2005). For example, finding biocontrol agents against foliar-specific pathogens would likely require screening microbes that can colonize the phyllosphere. Culturing phyllosphere-associated microbes from tomato (Enya et al., 2007) and wheat (Yoshida et al., 2012) has resulted in the identification of potential biocontrol microorganisms for foliar pathogens. Likewise, screening for biocontrol against post-harvest diseases would require identifying microbes that effectively protect the harvested crop (Janisiewicz and Korsten, 2002).

Successful candidate identification starts with a suitable population of microbes to be evaluated. While screening processes are becoming more robust and generating higher throughput, less than 1% of candidate microbes make successful products (Bailey and Falk, 2011). The generation of large microbe collections, both through targeted and broad sampling techniques, is required for identifying biocontrol candidates. One example, Trichobank^TM^, is a fungal culture collection of >2000 isolates of 21 *Trichoderma* spp. (Stewart et al., 2010). This collection has been successfully used to develop biocontrol agents like Sentinel^TM^ for the control of gray mold of grapes caused by *Botrytis cinerea*. In the case of microbe databases like Trichobank^TM^, information on the isolates within the collection is matched to a desired set of biocontrol capabilities based on pathogen targets, host plants, mode of action, and environmental niche (Glare et al., 2012). This subset of selected isolates is then subjected to a series of standardized bioassays to establish biocontrol efficacy and field performance capability. It is worth noting here that biocontrol efficacy in a field setting is key for adoption and implementation of microbial products. While many biocontrol agents were identified and/or validated through *in vitro* screens, caution should be taken when assuming correlation between *in vitro* inhibition and field performance (Burr et al., 1996; Milus and Rothrock, 1997; Fravel, 2005). Screening strategies can follow varied approaches, but the desired outcome is the same in identifying efficacious, environmentally safe, and cost-effective biocontrol agents (Köhl et al., 2011; Ravensberg, 2011).

## Success of A Product

The success of agricultural microbial products, whether biofertility or biocontrol, is rarely due to just one attribute, but instead is generally due to a number of factors (Ravensberg, 2011). Gelernter and Lomer (2000) suggest a framework for evaluating successful biocontrol products, but here we improve on these criteria to include all microbial products. Aside from technical efficacy, or the ability to improve yield or reduce crop damage, successful products meet two or more of the following conditions.

### Efficacy

The most important factor for a successful product is the ability to increase or protect yield. This is obviously the most important goal and a given factor in combination with other factors mentioned below for overall product success. However, efficacy in the laboratory and/or greenhouse does not always translate to field success (Nicot et al., 2011). Whipps (2001) stated “The key to achieving successful, reproducible biological control is the gradual appreciation that knowledge of the ecological interactions taking place in soil and root environment is required to predict the conditions under which biocontrol can be achieved.” Nicot et al. (2011) suggests that the success gap between lab and field efficacy can be improved by understanding of in-field mode of action. Although they specifically refer to biocontrol microbes, this same principle applies to biofertility products as well. Efficacy data that do not account for ecological interactions in a complex microbe-plant field ecosystem including at least some of the factors discussed below risks failure (Fravel, 2005). In many cases biocontrol microbial products are included as a part of an integrated pest management program (Chandler et al., 2011).

In addition to in field-efficacy, there are often efficacy challenges that arise with scaling production for widespread distribution. Some of the challenges described by Takors (2012) include the genetic stability of the strain and the impact of mutation, viruses, and phase variation, as well as other chemical and physical factors associated with going from bench-top to industrial scale bioreactors.

### Versatility

Plants may recruit specific microbes based on their development and environment, responding to stresses or nutrient availability (Smalla et al., 2006; Hartmann et al., 2009). The effectiveness of microbial strains colonizing the plant are impacted by a number of biotic and abiotic factors (Barea, 2015), including the previous cropping history (Berg and Smalla, 2009; Peiffer et al., 2013), suggesting that microbial compositions of the soil are modulated by changes in cropping practices. The ability to colonize is also impacted by genotype of the plant, showing variations in community structure between variants in the same species (Siciliano and Germida, 1999; Briones et al., 2002). Specific plant exudates in the form of volatile organic compounds, carbon sources or organic acids encourage colonization and growth of a relatively narrow group of organisms (Sloan and Lebeis, 2015). For example, studies on *Arabidopsis* demonstrate not only bacteria-specific responses to targeted exudates like malic acid (Rudrappa et al., 2008), but also community responses over time due to development stage of the plant (Chaparro et al., 2014). In addition to targeted strains and temporal development of colonization, strains must also associate with the appropriate root architecture of the plant, whether by interaction with receptors on the surface of the roots or by maintenance of cell numbers within the rhizosphere influenced by the plant (Compant et al., 2010). These factors allow for selective colonization of specific microorganisms and promote diversity of the community to fit the functional needs of the plant (Mendes et al., 2015). Improving microbial support for a crop requires understanding the needs of the plant, in combination with the composition of the soil and the surrounding communities to best determine the products that will benefit the crop. However, it should be noted that there is often an ecological trade-off when selecting for a specific trait in a microbial strain. For example Ehinger et al. (2014) explored the relationship between *Bradyrhizobium* and either specialized or generalized hosts and found a trade-off in host range and efficacy. Conversely, selecting for or developing strains that are specialists and highly effective in desired traits such as biocontrol or host interaction can result in loss of fitness (Kassen, 2002).

Biocontrol microbial strains are often highly targeted to specific species of pests (Nicot et al., 2011), so farmers may need to apply different products to control multiple pest species. Relevant narrowed spectrum, short-lasting, slowing-kill microbial based products are big hurdles for successful product commercialization. For example, the fungus *Colletotrichum gloeosporioides* f. sp. *malvae* was discovered to cause seedling blight on round-leaved mallow plants being grown in weed control trials in Saskatchewan (Harding and Raizada, 2015). However, the diversity of weeds in the field combined with its narrow host range have limited its usage in the market. Since ecological interactions are so important to in-field efficacy, organisms that have greater versatility will have improved efficacy over a number of different field conditions. This versatility includes interaction with different hosts and different pathogens and will be an ongoing process as pathogens continuously evolve to circumvent plant defenses and overcome biocontrol mechanisms (Brockhurst and Koskella, 2013; Zhan et al., 2014).

### Practicality

Another important factor in the success of an inoculant or biocontrol product is practicality for both the producer and the consumer. The product must ideally have a low barrier to adoption and be compatible with the farmer’s equipment and production practices.

Mass production of the microbe responsible for improving crop yield is one of the prime requirements for commercialization (Moosavi and Zare, 2015). *Pasteuria* is a good case study of a product that in the past was not very practical from an industrial perspective due to difficulties with mass production. *Pasteuria* was originally described from water fleas over 100 years ago, however, cultivation efforts were unsuccessful (Metchnikoff, 1888). Nearly two decades later, Cobb (1906) discovered these organisms infecting a nematode. *Pasteuria* species are able to effectively parasitize different developmental stages of nematodes (Chen and Dickson, 1998), but for over a century commercialization of *Pasteuria* was limited due to the inability to mass produce spores as a product. *Pasteuria penetrans* is an obligate parasite of *Meloidogyne* species, which are obligate plant parasites (Davies, 2009). Until recently, harvesting spores for commercial product required extracting spores from infected nematodes extracted from infected plants and was not an ideal system for mass production. It is currently a commercial product Clarivar^®^ (Syngenta^®^^13^).

Many farmers perceive inoculants and biocontrol microbial products as more costly and less effective than traditional agrochemicals. For example, microbial biocontrol strains are not always a quick acting option: they often work by suppressing pest populations through slower processes rather than killing on contact which may allow crop damage to continue for some amount of time. In some cases, to use biocontrol strains effectively, growers need to identify and know a great deal about the lifecycle of the pest or pathogen they are trying to control and understand the timing and appropriate conditions for application of the product. More outreach is needed between industrial or technical specialists and the agricultural community to help growers accustomed to broad-spectrum agrochemicals integrate inoculants and biocontrol microbial products into their cropping systems.

### Delivery

Appropriate formulation is required for a high quality product. Since microbial products are often stored under less than optimum conditions (e.g., high temperature, light exposure, high humidity), they must have an extended shelf life and the microorganism needs to be either robust or well protected to be able to survive under harsh conditions. Good formulation will also provide optimal conditions to enhance microorganism life on roots or on leaves to obtain optimal benefits after application to the target plants. To be widely adopted by farmers, an inoculant or biocontrol product must be cost effective and easy to apply, ensuring that the microorganisms are delivered to the target plant in the most appropriate manner and form. Formulation of inoculants and biocontrol products is a crucial issue but little research has been conducted on this subject. For some strains, particularly gram positive spore formers, formulation and long-term stability methods are much more developed than for gram negative strains. A literature survey by Xavier et al. (2004) showed that since the 1980s, most rhizobial research focused on the bacterial genetics and physiology and less than 1% of research articles on rhizobia have focused on formulation aspects of products. However, there is a real need for improved formulations of products, to create and commercialize new microbial products that will be more effective, stable, and higher quality to meet farmers’ needs.

Formulation of products by adding compounds to active ingredients can improve field performance, shelf life, and stability (Warrior et al., 2002; Leggett et al., 2011; Ravensberg, 2011), ultimately reducing variability. Formulation allows for several functional goals including safety, effective application, and enhanced persistence (Ravensberg, 2011). A lack of published research in this area is likely indicative of protection through intellectual property, like trade secrets, which is often necessary to protect investments in product development. Industry investments in current and future technologies will be critical in formulating novel products. One example of formulation utility is around microbes that do not form spores (e.g., gram negatives) or microbes that are highly sensitive to desiccation and temperature extremes. *Serratia entomophila* is the active ingredient in BioShield^®^, an insect biocontrol agent (Glare et al., 2012). New formulation techniques have reportedly allowed for stabilization of BioShield^®^ to extend shelf life to more than 6 months without loss of viability (Swaminathan and Jackson, 2011). In addition, formulation additives like diluents and oils have been used successfully for *Metarhizium acridium* products, enhancing fungal spore attachment and infection in target insects (Hunter, 2010).

The microbial ecology of biocontrol agents has been shown to indicate whether they are rhizosphere or phyllosphere competent (Kamilova et al., 2005; Bruck, 2010; Vorholt, 2012). Formulation technologies can therefore be used to improve delivery, colonization, germination, and establishment of microbes in those particular zones. Seed coating with microbes can provide an inexpensive option for targeted delivery, but improvements still need to be made in coating materials, microbe and chemistry compatibility and application technology, especially when considering the diverse requirements of biological organisms (Glare et al., 2012). One of these requirements is water availability, which can have profound influence on survival of bio-products (Connick et al., 1996). Dry or desiccated products weigh less, are more cost effective to ship, and have a lower risk of contamination. This type of formulation may be amenable to microbes that produce stable storage structures like spores, but non-spore producers likely require different formulation strategies.

Closely tied to formulation parameters is the actual delivery system used to apply beneficial microbes in an agriculture setting. A delivery system targeting precise timing and specific sites can greatly improve both bioinoculant and biocontrol product efficacy, persistence, and cost-effectiveness. Delivery presents a major challenge to industry in part because it requires mass production, formulation, and application of biocontrol microbes and/or their bioactive compounds (Ravensberg, 2011; Glare et al., 2012). As previously mentioned, the biology of the microbe may dictate the best avenue for delivery, leading to decisions of application site (e.g., seed, foliar, root) and timing. Researchers are looking beyond traditional seed coats or foliar sprays and investigating aspects of timing and treatment location. Varied spray schedules of *Trichoderma* biocontrol strains were used to control gray mold and anthracnose in strawberries (Freeman et al., 2004). Continuous application of the *Pseudomonas putida* in low concentrations through irrigation water resulted in soil populations similar to a single application at a 10-fold higher concentration (Steddom and Menge, 2001). This suggests that targeted delivery systems (site and timing) can result in field efficacy in a cost-saving manner.

### Persistence

Some of the issues associated with failure of microbial products involve the timing of the application of the product in the field (Chutia et al., 2007). Microbial products tend to act on more specific targets and have a shorter shelf and sometimes active life than chemical fertilizer/pesticides (van Lenteren, 2012). The combination of selectivity of the microbial strain to host or target and lack of persistence often results in inconsistent field data (Nicot et al., 2011). For example, Bt toxin proteins are degraded very quickly when they are exposed to sunlight. Bt-based microbial products often need multiple applications and result in high cost. In other cases, the efficacy of a product presents a tradeoff between immediate short-lived impact and persistence in the environment (Barea, 2015). The persistence of strains varies greatly in the environment. Some strains such as *Trichoderma harzianum* and *Bacillus amyloliquefaciens* FZB42 decrease below detectable limits within a few weeks of application (Papavizas, 1982; Kröber et al., 2014), whereas other strains such as *Rhizobium phaseoli* and *Bradyrhizobium japonicum* will persist indefinitely, but at a lower abundance than is required for efficacy (Robert and Schmidt, 1983; Narożna et al., 2015). Some products may be formulated to successfully enable persistence of the product long enough to show activity due to compatibility between a strain and the environment if they can occupy a niche or colonize before competitors show up (Verbruggen et al., 2012) impacting community assembly in the rhizosphere (Nemergut et al., 2013). In cases where the biological product does not readily colonize the rhizo/phyllosphere, compatibility and niche space in the environment will severely impact efficacy.

### Commercial Viability

High cost associated with production is another obstacle for success of developing a biological product. For example, AMF products generally contain spores, colonized roots, hyphae segments, or a mixture of the three (Dalpé and Monreal, 2004), and a wide range of carriers can be used (peat, compost, vermiculite, perlite, sand). Because AMF are obligate symbionts in nature, their proliferation and high-scale production require more specific skills and infrastructure. First attempts in AMF cultures used the pot-culture methods. Colonized root segments or spores of well-known AMF species are used to inoculate young seeds in a fresh sterile substrate. Plants are grown in pots, bags, or beds and the AMF colonize roots and substrate as the host develops, leading to a high concentration of AMF spores and colonized roots. Spores and roots obtained can then be used for commercial product preparation or to inoculate a new batch of sterile substrate. This kind of production method faced some difficulties such as uniformity of spores from batch to batch, production space requirements, and quality variation. In addition to production costs and return on investment for farmers, economic aspects of agricultural microbial products include market size and value (Nicot et al., 2011).

### Regulations

Regulatory frameworks and product registrations are used worldwide to guide the commercial development of microbial products. When developing new microbial products, the requisite regulatory framework varies by country, the product’s characteristics, and its intended usage. These national and international regulations must be taken into account during every part of the product development cycle, including its earliest stages, as certain regulations also outline where natural microbes can and cannot be harvested. Interestingly, the regulations pertaining to inoculants and biocontrol strains, while similar, may differ in certain parts of the world. Nevertheless, regulatory cycles for the development of new bioinoculants and biocontrol products are generally streamlined and well-articulated. As a result, microbial products are an appealing and cost-effective choice when taking an integrated, systems-level approach toward crop productivity and agricultural pest management.

## Conclusion

Microbial products to improve crop yields and health are readily available commercially, and their quality as well as efficacy has improved considerably over the past decade. The field performance of these products continues to be enhanced as major agricultural companies commit substantial research revenues to discovery and development of new products. Determining the appropriate microbial products for the functional needs of each crop will require input from both farmers and researchers. Soil type, microbiome, environmental conditions, pest presence and cropping system are all factors that could influence the benefit that a microbe may provide. The crop being planted is another key consideration, as many plants colonized by specific bacteria are unable to maintain high populations when other crops are planted. Further exploration into the mechanisms and specificity of plant growth promotion from key microorganisms will refine their specific use and maximize the potential inherent in the microbiome of plants and soils. In this regard, recently published studies (Agler et al., 2016; van der Heijden and Hartmann, 2016) revealed that the complex, interconnected microbial communities associated with plants harbor discrete keystone species, termed “microbial hubs” that play a critical role in mediating communications between the plant and its microbiome. Clearly, the ability to influence these functions for more efficacious biofertility and biocontrol applications is an area that will receive much attention.

Increased understanding of the impact microorganisms play in the growth and development of crops is key to future development of microbial products. In-depth studies into the effects of consortia and bacterial community structure on crop development will continue to expand our knowledge of the necessary effects the microbial community has on plants. Further examination of responses between target crops and microbes will better determine the specific signals that recruit or prevent colonizing microorganisms of critical food crops. These areas of research will result in a better understanding of the complex associations between the microbes in the soil and critical crops, a necessary step in providing farmers the tools necessary to continue feeding the planet in a sustainable manner.

## Author Contributions

All authors listed, have made substantial, direct and intellectual contribution to the work, and approved it for publication.

## Conflict of Interest Statement

The authors are employed by Novozymes.

The reviewer RD and handling Editor declared their shared affiliation, and the handling Editor states that the process nevertheless met the standards of a fair and objective review.

## Footnotes

1 http://www.monsantobioag.com/global/us/Products/Documents/Labels/Optimize_200_LiquidSoybean_Extended_Label.pdf.

2 http://www.abm1st.com/crops-products/soybeans/excalibre-sa/

3 http://www.mycogold.com/.

4 http://www.terramaxag.com/products/micro-az-st-dry/

5 http://www.agriculturesolutions.ca/bio-n-azotobacter-inoculants

6 http://jhbiotech.com/docs/Flyer-Mycormax.pdf

7 https://bio-organics.com/product/endomycorrhizal-inoculant/

8 http://mycorrhizae.com

9 http://www.microbesmart.com.au/index.php/what-is-vam

10 http://www.monsantobioag.com/global/us/harvest/pages/corn.aspx.

11 http://www.abm1st.com/crops-products/soybeans/excalibre-sa/

12 http://mycorrhizae.com/.

13 http://www.syngentacropprotection.com/clariva-complete-beans-seed-treatment

## References

[B1] Agler M. T. Ruhe J. Kroll S. Morhenn C. Kim S. T. Weigel D. (2016). Microbial hub taxa link host and abiotic factors to plant microbiome variation. *PLoS Biol.* 14:e1002352 10.1371/journal.pbio.1002352 PMC4720289 26788878

[B2] Akladious S. A. Abbas S. M. (2012). Application of *Trichoderma* T22 as a biofertilizer supporting maize growth. *Afr. J. Biotechnol.* 11 8672–8683.

[B3] Alexander M. (1984). *Biological Nitrogen Fixation: Ecology, Technology & Physiology.* New York, NY: Plenum Press.

[B4] Alexandratos N. Bruinsma J. (2012). *World Agriculture Towards 2030/2050: The 2012 Revision: ESA Working Paper No. 12-03* (Rome: FAO), 1–146.

[B5] Bailey K. Falk S. (2011). Turning research on microbial bioherbicides into commercial products – A phoma story. *Pest Technol.* 5 73–79.

[B6] Bardin M. Ajouz S. Comby M. Lopez-Ferber M. Graillot B. Siegwart M. (2015). Is the efficacy of biological control against plant diseases likely to be more durable than that of chemical pesticides? *Front. Plant Sci.* 6:566 10.3389/fpls.2015.00566 PMC4515547 26284088

[B7] Barea J. M. (2015). Future challenges and perspectives for applying microbial biotechnology in sustainable agriculture based on a better understanding of plant-microbiome interactions. *J. Soil Sci. Plant Nutr.* 15 261–282.

[B8] Basf-Corporation (2015). *Vault^®^ HP Plus Integral^®^ Technical Bulletin.* Available at: http://agproducts.basf.us/products/research-library/vault-hp-plus-integral-for-soybean-tech-bulletin.pdf

[B9] Bashan Y. De-Bashan L. E. Prabhu S. R. Hernandez J.-P. (2014). Advances in plant growth-promoting bacterial inoculant technology: formulations and practical perspectives (1998–2013). *Plant Soil* 378 1–33. 10.1007/s11104-013-1956-x

[B10] Bashan Y. Holguin G. (1997). *Azospirillum* – plant relationships: environmental and physiological advances (1990 – 1996). *Can. J. Microbiol.* 43 103–121. 10.1139/m97-015 15467782

[B11] Bashan Y. Ream Y. Levanony H. Sade A. (1988). Nonspecific responses in plant growth, yield, and root colonization of noncereal crop plants to inoculation with *Azospirillum brasilense* Cd. *Can. J. Bot.* 67 1317–1324. 10.1139/b89-175

[B12] Baulcombe D. Crute I. Davies B. Dunwell J. Gale M. Jones J. (2009). *Reaping the Benefits: Science and the Sustainable Intensification of Global Agriculture.* The Royal Society Report London: The Royal Society.

[B13] Berg G. (2009). Plant-microbe interactions promoting plant growth and health: perspectives for controlled use of microorganisms in agriculture. *Appl. Microbiol. Biotechnol.* 84 11–18. 10.1007/s00253-009-2092-7 19568745

[B14] Berg G. Smalla K. (2009). Plant species and soil type cooperatively shape the structure and function of microbial communities in the rhizosphere. *FEMS Microbiol. Ecol.* 68 1–13. 10.1111/j.1574-6941.2009.00654.x 19243436

[B15] Beuerlein J. (2008). *Why I Inoculate Soybeans.* C.O.R.N. Newsletter 2008-06 Columbus, OH: Ohio State University Extension.

[B16] Bhattacharyya P. N. Jha D. K. (2012). Plant growth-promoting rhizobacteria (PGPR): emergence in agriculture. *World J. Microbiol. Biotechnol.* 28 1327–1350. 22805914 10.1007/s11274-011-0979-9

[B17] Briones A. M. Okabe S. Umemiya Y. Ramsing N.-B. Reichardt W. Okuyama H. (2002). Influence of different cultivars on populations of ammonia-oxidizing bacteria in the root environment of rice. *Appl. Environ. Microbiol.* 68 3067–3075. 10.1128/AEM.68.6.3067-3075.2002 12039768 PMC123923

[B18] Brockhurst M. A. Koskella B. (2013). Experimental coevolution of species interactions. *Trends Ecol. Evol.* 28 367–375. 10.1016/j.tree.2012.04.010 23523051

[B19] Bruck D. J. (2010). Fungal entomopathogens in the rhizosphere. *Biocontrol* 55 103–112. 10.1007/s10526-009-9236-7

[B20] Bruinsma J. (2009). “The resource outlook to 2050: by how much do land, water and crop yields need to increase by 2050?” in *Proceedings of the FAO Expert Meeting on How to Feed the World in 2050* (Rome: FAO), 1–33.

[B21] Burr T. Matteson M. Smith C. Corral-Garcia M. Huang T.-C. (1996). Effectiveness of bacteria and yeasts from apple orchards as biological control agents of apple scab. *Biol. Control* 6 151–157. 10.1006/bcon.1996.0019

[B22] Catroux G. Hartmann A. Revellin C. (2001). Trends in rhizobial inoculant production and use. *Plant Soil* 230 21–30. 10.1023/A:1004777115628

[B23] Chandler D. Bailey A. S. Tatchell G. M. Davidson G. Greaves J. Grant W. P. (2011). The development, regulation and use of biopesticides for integrated pest management. *Philos. Trans. R. Soc. Lond. B Biol. Sci.* 366 1987–1998. 10.1098/rstb.2010.0390 21624919 PMC3130386

[B24] Chaparro J. M. Badri D. V. Vivanco J. M. (2014). Rhizosphere microbiome assemblage is affected by plant development. *ISME J.* 8 790–803. 10.1038/ismej.2013.196 24196324 PMC3960538

[B25] Chen Z. X. Dickson D. W. (1998). Review of *Pasteuria penetrans*: biology, ecology, and biological control potential. *J. Nematol.* 30 313–340. 19274225 PMC2620303

[B26] Chutia M. Mahanta J. J. Bhattacheryya N. Bhuyan M. Boruah P. Sarma T. C. (2007). Microbial herbicides for weed management: prospects, progress and constraints. *Plant Pathol. J.* 6 210–218. 10.3923/ppj.2007.210.218

[B27] Cobb N. A. (1906). “Free living nematodes inhabiting the soil about the roots of cane, and their relation to root diseases,” in *Fungus Maladies of the Sugar Cane: With Notes on Associated Insects and Nematodes* (Honolulu, HI: Hawaiian Gazette Co. Ltd), 163–195.

[B28] Compant S. Clément C. Sessitsch A. (2010). Plant growth-promoting bacteria in the rhizo- and endosphere of plants: their role, colonization, mechanisms involved and prospects for utilization. *Soil Biol. Biochem.* 42 669–678. 10.1016/j.soilbio.2009.11.024

[B29] Connick W. Jr. Daigle D. Boyette C. Williams K. S. Vinyard B. T. Quimby P. C. Jr. (1996). Water activity and other factors that affect the viability of *Colletotrichum truncatum* conidia in wheat flour-kaolin granules (‘Pesta’). *Biocontrol Sci. Technol.* 6 277–284. 10.1080/09583159650039467

[B30] Copping L. G. (2009). *Manual of Biocontrol Agents*, 4th Edn Alton: British Crop Protection Council, 1350.

[B31] CPL (2006). *Biopesticides 2007 Vol. 4 Europe: The Biopesticide Market.* Wallingford, CT: CPL Business Consultants.

[B32] Cunningham J. E. Kuiack C. (1992). Production of citric and oxalic acids and solubilization of calcium phosphate by *Penicillium bilaii*. *Appl. Environ. Microbiol.* 58 1451–1458. 1622211 10.1128/aem.58.5.1451-1458.1992 PMC195625

[B33] Dalpé Y. Monreal M. (2004). Arbuscular mycorrhiza inoculum to support sustainable cropping systems. *Crop Manag.* 3 10.1094/CM-2004-0301-09-RV

[B34] Davies K. G. (2009). Understanding the interaction between an obligate hyperparasitic bacterium, *Pasteuria penetrans* and its obligate plant-parasitic nematode host, *Meloidogyne* spp. *Adv. Parasitol.* 68 211–245. 10.1016/S0065-308X(08)00609-X 19289196

[B35] de Bruijn F. (2013). *Molecular Microbial Ecology of the Rhizosphere.* Hoboken, NJ: Wiley-Blackwell.

[B36] Deaker R. Roughly R. J. Kennedy I. R. (2004). Legume seed inoculation technology - a review. *Soil Biol. Biochem.* 36 1275–1288. 10.1016/j.ram.2015.06.006

[B37] Diaz-Zorita M. R. Balińa M. Micucci F. G. Lastra V. D. (2012). “Field inoculation of cereals grain crops with *Azospirillum brasilense* in the pampas, Argentina,” in *Poster Presentation Visions for a sustainable planet - ASA, CSSA and SSSA International Annual Meetings*, (Cinncinnati, OH: Soil Science Society of America).

[B38] Ehinger M. Mohr T. J. Starcevich J. B. Sachs J. L. Porter S. S. Simms E. L. (2014). Specialization-generalization trade-off in a *Bradyrhizobium* symbiosis with wild legume hosts. *BMC Ecol.* 14:1 10.1186/1472-6785-14-8 PMC4021497 24641813

[B39] Ehrlich P. R. Ehrlich A. H. (1990). *The Population Explosion.* London: Hutchinson.

[B40] Elad Y. (2003). Biocontrol of foliar pathogens: mechanisms and application. *Commun. Agric. Appl. Biol. Sci.* 68 17–24. 15149089

[B41] Enya J. Shinohara H. Yoshida S. Tsukiboshi T. Negishi H. Suyama K. (2007). Culturable leaf-associated bacteria on tomato plants and their potential as biological control agents. *Microb. Ecol.* 53 524–536. 10.1007/s00248-006-9085-1 17356949

[B42] Erisman J. W. Sutton M. A. Galloway J. Klimont Z. Winiwarter W. (2008). How a century of ammonia synthesis changed the world. *Nat. Geosci.* 1 636–639. 10.1038/ngeo325

[B43] Erlacher A. Cardinale M. Grosch R. Grube M. Berg G. (2014). The impact of the pathogen *Rhizoctonia solani* and its beneficial counterpart *Bacillus amyloliquefaciens* on the indigenous lettuce microbiome. *Front. Microbiol.* 5:175 10.3389/fmicb.2014.00175 PMC4001036 24795707

[B44] Fan B. Chen X. H. Budiharjo A. Bleiss W. Vater J. Borriss R. (2011). Efficient colonization of plant roots by the plant growth promoting bacterium *Bacillus amyloliquefaciens* FZB42 engineered to express green fluorescent protein. *J. Biotechnol.* 151 303–311. 10.1016/j.jbiotec.2010.12.022 21237217

[B45] Fravel D. R. (2005). Commercialization and implementation of biocontrol. *Annu. Rev. Phytopathol.* 43 337–359. 10.1146/annurev.phyto.43.032904.092924 16078888

[B46] Freeman S. Minz D. Kolesnik I. Barbul O. Zveibil A. Maymon M. (2004). *Trichoderma* biocontrol of *Colletotrichum acutatum* and *Botrytis cinerea* and survival in strawberry. *Eur. J. Plant Pathol.* 110 361–370. 10.1023/B:EJPP.0000021057.93305.d9

[B47] Frost and Sullivan (2009). *North American & Western European Biopesticides Market.* Report M472 Chennai: Frost and Sullivan.

[B48] Gelernter W. D. Lomer C. J. (2000). “Success in biological control of above-ground insects by pathogens,” in *Biological Control: Measures Of Success*, eds Gurr G. Wratten S. (Dordrecht: Kluwer), 297–322.

[B49] Gilbert N. (2009). Environment: the disappearing nutrient. *Nature* 461 716–718. 10.1038/461716a 19812648

[B50] Glare T. Caradus J. Gelernter W. Jackson T. Keyhani N. Köhl J. (2012). Have biopesticides come of age? *Trends Biotechnol.* 30 250–258. 10.1016/j.tibtech.2012.01.003 22336383

[B51] Grooms L. (2008). *Better Inoculants. Farm Industry News.* Available at: http://farmindustrynews.com/soybean-varieties/better-inoculants

[B52] Grosch R. Dealtry S. Schreiter S. Berg G. Mendonça-Hagler L. Smalla K. (2012). Biocontrol of *Rhizoctonia solani*: complex interaction of biocontrol strains, pathogen and indigenous microbial community in the rhizosphere of lettuce shown by molecular methods. *Plant Soil* 361 343–357. 10.1007/s11104-012-1239-y

[B53] Hardin G. (1998). The feast of Malthus: living within the limits. *Soc. Contract* 181–187.

[B54] Harding D. P. Raizada M. N. (2015). Controlling weeds with fungi, bacteria and viruses: a review. *Front. Plant Sci.* 6:659 10.3389/fpls.2015.00659 PMC4551831 26379687

[B55] Hartmann M. Lee S. Hallam S. J. Mohn W. W. (2009). Bacterial, archaeal and eukaryal community structures throughout soil horizons of harvested and naturally disturbed forest stands. *Environ. Microbiol.* 11 3045–3062. 10.1111/j.1462-2920.2009.02008.x 19659501

[B56] Hodge A. Storer K. (2015). Arbuscular mycorrhiza and nitrogen: implications for individual plants through to ecosystems. *Plant Soil* 386 1–19. 10.1007/s11104-014-2162-1

[B57] Hunter D. M. (2010). Credibility of an IPM approach for locust and grasshopper control: the Australian example^∗^ *J. Orthoptera Res*. 19 133–137. 10.1665/034.019.0108

[B58] Janisiewicz W. J. Korsten L. (2002). Biological control of postharvest diseases of fruits. *Annu. Rev. Phytopathol.* 40 411–441. 10.1146/annurev.phyto.40.120401.130158 12147766

[B59] Johnson T. D. (2013). *Use of Synergistic Microorganisms and Nutrients to Produce Signals that Facilitate the Germination and Plant Root Colonization of Mycorrhizal Fungi in Phosphorus Rich Environments. US Patent Application 201 A0276491.* Washinton, DC: United States Patent and Trademark Office.

[B60] Johnson T. D. (2015). *Use of Synergistic Microorganisms and Nutrients to Produce Signals that Facilitate the Germination and Plant Root Colonization of Mycorrhizal Fungi in Phosphorus Rich Environments. Us Patent Application 201 A0329431.* Washinton, DC: United States Patent and Trademark Office.

[B61] Junaid J. M. Dar N. A. Bhat T. A. Bhat A. H. Bhat M. A. (2013). Commercial biocontrol agents and their mechanism of action in the management of plant pathogens. *Int. J. Mod. Plant Anim. Sci.* 1 39–57. 10.3109/07388551.2010.487258

[B62] Kamilova F. Validov S. Azarova T. Mulders I. Lugtenberg B. (2005). Enrichment for enhanced competitive plant root tip colonizers selects for a new class of biocontrol bacteria. *Environ. Microbiol.* 7 1809–1817. 10.1111/j.1462-2920.2005.00889.x 16232295

[B63] Kassen R. (2002). The experimental evolution of specialists, generalists, and the maintenance of diversity. *J. Evol. Biol.* 15 173–190. 10.1098/rspb.2015.1932

[B64] Kiewnick S. (2007). Practicalities of developing and registering microbial biological control agents. *CAB Rev.* 2 1–11. 10.1079/PAVSNNR20072013

[B65] Köhl J. Postma J. Nicot P. Ruocco M. Blum B. (2011). Stepwise screening of microorganisms for commercial use in biological control of plant-pathogenic fungi and bacteria. *Biol. Control* 57 1–12. 10.1016/j.biocontrol.2010.12.004

[B66] Kröber M. Wibberg D. Grosch R. Eikmeyer F. Verwaaijen B. Chowdhury S. P. (2014). Effect of the strain *Bacillus amyloliquefaciens* FZB42 on the microbial community in the rhizosphere of lettuce under field conditions analyzed by whole metagenome sequencing. *Front. Microbiol.* 5:252 10.3389/fmicb.2014.00252 PMC4033844 24904564

[B67] Lamdan N.-L. Shalaby S. Ziv T. Kenerley C. M. Horwitz B. A. (2015). Secretome of *Trichoderma* interacting with maize roots: role in induced systemic resistance. *Mol. Cell. Proteomics* 14 1054–1063. 10.1074/mcp.M114.046607 25681119 PMC4390251

[B68] Larkin R. P. Fravel D. R. (2002). Effects of varying environmental conditions on biological control of *Fusarium* wilt of tomato by nonpathogenic *Fusarium* spp. *Phytopathology* 92 1160–1166. 10.1094/PHYTO.2002.92.11.1160 18944240

[B69] Leggett M. Gleddie S. Holloway G. (2001). “Phosphate-solubilizing microorganisms and their use,” in *Plant Nutrient Acquisition*, eds Ae N. Arihara J. Okada K. Srinivasan A. (Tokyo: Springer-Verlag), 299–318.

[B70] Leggett M. Leland J. Kellar K. Epp B. (2011). Formulation of microbial biocontrol agents–an industrial perspective. *Can. J. Plant Pathol.* 33 101–107. 10.1080/07060661.2011.563050

[B71] Leggett M. Newlands N. K. Greenshields D. West L. Inman S. Koivunen M. E. (2015). Maize yield response to a phosphorus-solubilizing microbial inoculant in field trials. *J. Agric. Sci.* 153 1464–1478. 10.1017/S0021859614001166 26500375 PMC4611360

[B72] Lehr P. (2010). *Biopesticides: The Global Market.* Report Code CHM029B London: BCC Research.

[B73] Lo C.-T. (1998). General mechanisms of action of microbial biocontrol agents. *Plant Pathol. Bull.* 7 155–166.

[B74] Mendes L. W. Tsai S. M. Navarrete A. A. Hollander M. Veen J. A. Kuramae E. E. (2015). Soil-borne microbiome: linking diversity to function. *Microb. Ecol.* 70 255–265. 10.1007/s00248-014-0559-2 25586384

[B75] Metchnikoff M. E. (1888). *Pasteuria ramosa* un prepresentant des bacteries a division longitudinale. *Ann. Inst. Pasteur.* 2 165–170.

[B76] Milus E. Rothrock C. (1997). Efficacy of bacterial seed treatments for controlling *Pythium* root rot of winter wheat. *Plant Dis.* 81 180–184. 10.1094/PDIS.1997.81.2.180 30870893

[B77] Molla A. H. Manjurul Haque M. Amdadul Haque M. Ilias G. N. M. (2012). *Trichoderma*-enriched biofertilizer enhances production and nutritional quality of tomato (*Lycopersicon esculentum* mill.) and minimizes NPK fertilizer use. *Agric. Res.* 1 265–272. 10.1007/s40003-012-0025-7

[B78] Monsanto BioAg Alliance (2015a). *Product Literature for JumpStart.* Available at: http://www.monsantobioag.com/global/ca/Products/Pages/jumpstart.aspx

[B79] Monsanto BioAg Alliance (2015b). *QuickRoots CORN Fact Sheet.* Available at: http://www.tjquickroots.com/doc/QuickRoots_CORN_Fact_Sheet_2012.pdf

[B80] Monsanto BioAg Alliance (2015c). *QuickRoots Soybean Technical Information.* Available at: http://www.monsantobioag.com/global/us/Products/Pages/QuickRoots-Soybean.aspx?gclid=CJag-I-WtcoCFU1gfgod10APjQ

[B81] Monsanto BioAg Alliance (2015d). *QuickRoots: Bacillus amyloliquefaciens and Trichoderma virens Based Inoculant for Corn (Product Information).* Available at: http://www.monsantobioag.com/global/us/Products/Pages/QuickRoots-Corn.aspx

[B82] Monsanto BioAg Alliance (2015e). *Monsanto BioAg Alliance – Product Literature for Optimize^®^ Soybean Liquid Inoculant.* Available at: http://monsantobioag.com/global/us/Products/Pages/Optimize-Soybean.aspx

[B83] Moosavi M. R. Zare R. (2015). “Factors affecting commercial success of biocontrol agents of phytonematodes,” in *Biocontrol Agents of Phytonematodes*, eds Askary T. H. Martinelli P. R. P. (Wallingford, CT: CABI Publishing), 423.

[B84] Nahas E. (1996). Factors determining rock phosphate solubilization by microorganisms isolated from soil. *World J. Microbiol. Biotechnol.* 12 567–572. 10.1007/BF00327716 24415416

[B85] Narożna D. Pudełko K. Króliczak J. Goliñska B. Sugawara M. Mkadrzak C. J. (2015). Survival and competitiveness of *Bradyrhizobium japonicum* strains 20 years after introduction into field locations in Poland. *Appl. Environ. Microbiol.* 81 5552–5559. 10.1128/AEM.01399-15 26048934 PMC4510166

[B86] Nemergut D. R. Schmidt S. K. Fukami T. O’Neill S. P. Bilinski T. M. Stanish L. F. (2013). Patterns and processes of microbial community assembly. *Microbiol. Mol. Biol. Rev.* 77 342–356. 10.1128/MMBR.00051-12 24006468 PMC3811611

[B87] Nicot P. Blum B. Kohl J. Ruocco M. (2011). “Perspectives for future research-and-development projects on biological control of plant pests and diseases,” in *Classical and Augmentative Biological Control Against Diseases and Pests: Critical Status Analysis and Review of Factors Influencing Their Success*, ed. Nicot P. (Zürich: IOB-International Organisation for Biological and Integrated Control of Noxious Animals and Plants), 68–70.

[B88] Nobbe F. Hiltner L. (1896). *Inoculation of the Soil for Cultivating. US Patent 570 813.* Washinton, DC: United States Patent and Trademark Office.

[B89] Oerke E. Dehne H. (2004). Safeguarding production - losses in major crops and the role of crop protection. *Crop Prot.* 23 275–285. 10.1016/j.cropro.2003.10.001

[B90] Okon Y. Labandera-Gonzales C. A. (1994). Agronomic applications of *Azospirillum*: an evaluation of 20 years worldwide field inoculation. *Soil Biol. Biochem.* 26 1591–1601. 10.1016/0038-0717(94)90311-5

[B91] Okon Y. Labandera-Gonzales C. Lage M. Lage P. (2015). “Agronomic applications of *Azospirillum* and other PGPR,” in *Biological Nitrogen Fixation*, 1st Edn, ed. De Bruijn F.J. (Hoboken, NJ: John Wiley & Sons, Inc.), 921–933.

[B92] Owen D. Williams A. P. Griffith G. W. Withers P. J. A. (2015). Use of commercial bio-inoculants to increase agricultural production through improved phosphrous acquisition. *Appl. Soil Ecol.* 86 41–54. 10.1016/j.apsoil.2014.09.012

[B93] Papavizas G. C. (1982). Survival of *Trichoderma harzianum* in soil and in pea and bean rhizospheres. *Phytopathology* 72 121–125. 10.1094/Phyto-72-121

[B94] Peiffer J. A. Spor A. Koren O. Jin Z. Tringe S. G. Dangl J. L. (2013). Diversity and heritability of the maize rhizosphere microbiome under field conditions. *Proc. Natl. Acad. Sci. U.S.A.* 110 6548–6553. 10.1073/pnas.1302837110 23576752 PMC3631645

[B95] Pelizza S. A. Scorsetti A. C. Fogel M. N. Pacheco-Marino S. G. Stenglein S. A. Cabello M. N. (2015). Compatibility between entomopathogenic fungi and biorational insecticides in toxicity against *Ronderosia bergi* under laboratory conditions. *Biocontrol* 60 81–91. 10.1007/s10526-014-9606-7

[B96] Porcel R. Aroca R. Ruiz-Lozano J. M. (2012). Salinity stress alleviation using arbuscular mycorrhizal fungi. A review. *Agron. Sustain. Dev.* 32 181–200. 10.1007/s13593-011-0029-x

[B97] Pradhan N. Sukla L. B. (2005). Solubilization of inorganic phosphates by fungi isolated from agriculture soil. *Afr. J. Biotechnol.* 5 850–854.

[B98] Rai M. K. (2006). *Handbook of Microbial Biofertilizers.* New York, NY: Food Products Press.

[B99] Rastogi G. Coaker G. L. Leveau J. H. (2013). New insights into the structure and function of phyllosphere microbiota through high-throughput molecular approaches. *FEMS Microbiol. Lett.* 348 1–10. 10.1111/1574-6968.12225 23895412

[B100] Ravensberg W. J. (2011). *A Roadmap to the Successful Development and Commercialization of Microbial Pest Control Products for Control of Arthropods.* Dordrecht: Springer Science & Business Media.

[B101] Reid A. Greene S. E. (2013). *How Microbes Can Help Feed the World.* Washington, DC: American Academy of Microbiology. 32687282

[B102] Richardson A. E. (1994). “Soil microorganisms and phosphorus availability,” in *Soil Biota: Management in Sustainable Farming Systems*, eds Pankhurst C. E. Doube B. M. Gupta V. V. S. R. Grace P. R. (East Melbourne, VIC: CSIRO), 50–62.

[B103] Richardson A. E. (2001). Prospects for using soil microorganisms to improve the acquisition of phosphorus by plants. *Austr. J. Plant Physiol.* 28 897–906.

[B104] Robert F. M. Schmidt E. L. (1983). Population changes and persistence of *Rhizobium phaseoli* in soil and rhizospheres. *Appl. Environ. Microbiol.* 45 550–556. 16346203 10.1128/aem.45.2.550-556.1983 PMC242322

[B105] Rodgers P. B. (1993). Potential of biopesticides in agriculture. *Pestic. Sci.* 39 117–129. 10.1002/ps.2780390205

[B106] Rudrappa T. Czymmek K. J. Paré P. W. Bais H. P. (2008). Root-secreted malic acid recruits beneficial soil bacteria. *Plant Physiol.* 148 1547–1556. 10.1104/pp.108.127613 18820082 PMC2577262

[B107] Sahoo R. K. Bhardwaj D. Tuteja N. (2013). “Biofertilizers: a sustainable eco-friendly agricultural approach to crop improvement,” in *Plant Acclimation to Environmental Stress*, eds Tuteja N. Gill S. S. (New York, NY: Springer Science & Business Media), 403–432.

[B108] Schachtman D. P. Reid R. J. Ayling S. M. (1998). Phosphorus uptake by plants: from soil to cell. *Plant Physiol.* 116 447–453. 10.1104/pp.116.2.447 9490752 PMC1539172

[B109] Scholz R. W. Ulrich A. E. Eilitta M. Roy A. (2013). Sustainable use of phosphorus: a finite resource. *Sci. Total Environ.* 46 799–803. 10.1016/j.scitotenv.2013.05.043 23769630

[B110] Shapiro-Ilan D. Gaugler R. (2002). Production technology for entomopathogenic nematdoes and their bacterial symbionts. *J. Ind. Microbiol. Biotechnol.* 28 137–146. 10.1038/sj.jim.7000230 12074087

[B111] Siciliano S. D. Germida J. J. (1999). Taxonomic diversity of bacteria associated with the roots of field-grown transgenic *Brassica napus* cv. Quest, compared to the non-transgenic *B. napus* cv. Excel and *B. rapa* cv. Parkland. *FEMS Microbiol. Ecol.* 29 263–272. 10.1111/j.1574-6941.1999.tb00617.x

[B112] Sloan S. S. Lebeis S. L. (2015). Exercising influence: distinct biotic interactions shape root microbiomes. *Curr. Opin. Plant Biol.* 26 32–36. 10.1016/j.pbi.2015.05.026 26116973

[B113] Smalla K. Sessitsch A. Hartmann A. (2006). The Rhizosphere: ‘soil compartment influenced by the root’. *FEMS Microbiol. Ecol.* 56:165 10.1111/j.1574-6941.2006.00148.x 16629746

[B114] Smith S. Habib A. Kang Y. Leggett M. Diaz-Zorita M. (2015). “LCO applications provide improved responses with legumes and nonlegumes,” in *Biological Nitrogen Fixation* Vol. 2 ed. de Bruijn F. (Hoboken, NJ: John Wiley & Sons, Inc), 1077–1086.

[B115] Smith S. E. Read D. J. (2008). *Mycorrhizal Symbiosis.* London: Academic Press.

[B116] Steddom K. Menge J. (2001). Evaluation of continuous application technology for delivery of the biocontrol agent *Pseudomonas putida* 06909-rif/nal. *Plant Dis.* 85 387–392. 10.1094/PDIS.2001.85.4.387 30831971

[B117] Stewart A. Ohkura M. Mclean K. (2010). “Targeted screening for microbial bioactivity,” in *Microbial Products: Exploiting Microbial Diversity for Sustainable Plant Production*, eds Zydenbos S. M. Jackson T. A. (Auckland: New Zealand Plant Protection Society Inc), 11–19.

[B118] Swaminathan J. A. Jackson T. A. (2011). Agent Stabilisation and Delivery Process and Product. NZ Patent 560574 27/04/11. Wellington: Intellectual Property Office of New Zealand.

[B119] Takors R. (2012). Scale-up of microbial processes: impacts, tools and open questions. *J. Biotechnol.* 160 3–9. 10.1016/j.jbiotec.2011.12.010 22206982

[B120] Terpolilli J. J. Hood G. A. Poole P. S. (2012). What determines the efficiency of N2-fixing *Rhizobium*-legume symbioses? *Adv. Microb. Physiol.* 60 325–389. 10.1016/B978-0-12-398264-3.00005-X 22633062

[B121] Timonin M. I. (1948). *Azotobacter* preparation (Azotogen) as a fertilizer for cultivated plants. *Soil Sci. Soc. Am. J.* 13 246–249. 10.2136/sssaj1949.036159950013000C0043x

[B122] Transparency Market Research (2014). *Biofertilizers (Nitrogen Fixing, Phosphate Solubilizing and Others) Market for Seed Treatment and Soil Treatment Applications – Global Industry Analysis, Size, Share, Growth, Trends and Forecast, 2013-2019.* Albany, NY: Transparency Market Research.

[B123] Ullah A. Mushtaq H. Ali H. Munis M. F. Javed M. T. Chaudhary H. J. (2015). Diazotrophs-assisted phytoremediation of heavy metals: a novel approach. *Environ. Sci. Pollut. Res. Int.* 22 2505–2514. 10.1007/s11356-014-3699-5 25339525

[B124] van der Heijden M. G. Hartmann M. (2016). Networking in the plant microbiome. *PLoS Biol.* 14:e1002378 10.1371/journal.pbio.1002378 PMC4752285 26871440

[B125] van Lenteren J. C. (2012). The state of commercial augmentative biological control: plenty of natural enemies, but a frustrating lack of uptake. *BioControl* 57 1–20. 10.1007/s10526-011-9395-1

[B126] Vance C. P. (2001). Symbiotic nitrogen fixation and phosphorus acquisition. plant nutrition in a world of declining renewable resources. *Plant Physiol.* 127 390–397. 10.1104/pp.010331 11598215 PMC1540145

[B127] Vargas L. Santa Brigida A. B. Mota Filho J. P. De Carvalho T. G. Rojas C. A. Vaneechoutte D. (2014). Drought tolerance conferred to sugarcane by association with *Gluconacetobacter diazotrophicus*: a transcriptomic view of hormone pathways. *PLoS ONE* 9:e114744 10.1371/journal.pone.0114744 PMC4260876 25489849

[B128] Verbruggen E. Van Der Heijden M. G. Weedon J. T. Kowalchuk G. A. Roeling W. F. (2012). Community assembly, species richness and nestedness of arbuscular mycorrhizal fungi in agricultural soils. *Mol. Ecol.* 21 2341–2353. 10.1111/j.1365-294X.2012.05534.x 22439851

[B129] Vorholt J. A. (2012). Microbial life in the phyllosphere. *Nat. Rev. Microbiol.* 10 828–840. 10.1038/nrmicro2910 23154261

[B130] Warrior P. Konduru K. Vasudevan P. (2002). “Formulation of biological control agents for pest and disease management,” in *Biological Control of Crop Diseases*, ed. Gnanamanickam S. S. (New York, NY: Marcel Dekker), 421–442.

[B131] Weidner S. Pühler A. Küster H. (2003). Genomics insights into symbiotic nitrogen fixation. *Curr. Opin. Biotechnol.* 14 200–205. 10.1016/S0958-1669(03)00022-3 12732321

[B132] Whipps H. M. (2001). Microbial interactions and biocontrol in the rhizosphere. *J. Exp. Bot.* 52 487–511. 10.1093/jexbot/52.suppl_1.487 11326055

[B133] Wilson E. O. (2003). *The Future of Life.* New York, NY: Vintage Books.

[B134] Xavier I. J. Holloway G. Leggett M. (2004). Development of rhizobial inoculant formulations. *Crop Manage.* 3 10.1094/CM-2004-0301-03-RV

[B135] Yoshida S. Ohba A. Liang Y.-M. Koitabashi M. Tsushima S. (2012). Specificity of *Pseudomonas* isolates on healthy and *Fusarium* head blight-infected spikelets of wheat heads. *Microb. Ecol.* 64 214–225. 10.1007/s00248-012-0009-y 22314388

[B136] Zahran H. H. (2009). “Enhancement of rhizobia-legumes sympbioses and nitrogen fixation for crops productivity improvement,” in *Microbial Strategies for Crop Improvement*, ed. Khan M. S. (Berlin: Springer-Verlag), 227–254.

[B137] Zhan J. Thrall P. H. Burdon J. J. (2014). Achieving sustainable plant disease management through evolutionary principles. *Trends Plant Sci.* 19 570–575. 10.1016/j.tplants.2014.04.010 24853471

